# Affinity Map of Bromodomain Protein 4 (BRD4) Interactions with the Histone H4 Tail and the Small Molecule Inhibitor JQ1*

**DOI:** 10.1074/jbc.M113.523019

**Published:** 2014-02-04

**Authors:** Marie Jung, Martin Philpott, Susanne Müller, Jessica Schulze, Volker Badock, Uwe Eberspächer, Dieter Moosmayer, Benjamin Bader, Norbert Schmees, Amaury Fernández-Montalván, Bernard Haendler

**Affiliations:** From the ‡ Global Drug Discovery, Bayer HealthCare, 13353 Berlin, Germany,; the § Institute of Chemistry and Biochemistry, Free University, 14195 Berlin, Germany, and; ¶ Structural Genomics Consortium and Target Discovery Institute, Nuffield Department of Clinical Medicine, University of Oxford, Oxford OX3 7DQ, United Kingdom

**Keywords:** Anticancer Drug, Cancer Therapy, Chromatin, Histones, Mutant, Acetylation, BET Family, BRD4, Bromodomain

## Abstract

Bromodomain protein 4 (BRD4) is a member of the bromodomain and extra-terminal domain (BET) protein family. It binds to acetylated histone tails via its tandem bromodomains BD1 and BD2 and forms a complex with the positive transcription elongation factor b, which controls phosphorylation of RNA polymerase II, ultimately leading to stimulation of transcription elongation. An essential role of BRD4 in cell proliferation and cancer growth has been reported in several recent studies. We analyzed the binding of BRD4 BD1 and BD2 to different partners and showed that the strongest interactions took place with di- and tetra-acetylated peptides derived from the histone 4 N-terminal tail. We also found that several histone 4 residues neighboring the acetylated lysines significantly influenced binding. We generated 10 different BRD4 BD1 mutants and analyzed their affinities to acetylated histone tails and to the BET inhibitor JQ1 using several complementary biochemical and biophysical methods. The impact of these mutations was confirmed in a cellular environment. Altogether, the results show that Trp-81, Tyr-97, Asn-140, and Met-149 play similarly important roles in the recognition of acetylated histones and JQ1. Pro-82, Leu-94, Asp-145, and Ile-146 have a more differentiated role, suggesting that different kinds of interactions take place and that resistance mutations compatible with BRD4 function are possible. Our study extends the knowledge on the contribution of individual BRD4 amino acids to histone and JQ1 binding and may help in the design of new BET antagonists with improved pharmacological properties.

## Teaser

**Background:** BRD4 is a reader of acetylated histones.

**Results:** Mutational analysis of BRD4 BD1 allowed the identification of three groups with different binding profiles.

**Conclusion:** Pro-82, Leu-94, Asp-145, and Ile-146 have a differentiated role in acetyl-lysine and inhibitor interaction.

**Significance:** Identification of residues essential for BRD4 function will guide the design of novel inhibitors.

## Introduction

Acetylation of histones is an essential post-translational modification governing gene expression (1, 2). It affects a number of lysine residues in all histones and serves as a docking site for epigenetic readers containing the evolutionarily highly conserved bromodomain module (3). A total of 61 bromodomains found in 46 different proteins have been identified in the human proteome (4). After the recognition of discrete patterns of acetylation marks, which together with neighboring amino acids, mediate binding selectivity, bromodomain-containing proteins engage in a variety of cellular functions. The four bromodomain and extra-terminal domain (BET)^3^ proteins each contain two related bromodomains named BD1 and BD2 in their N-terminal moiety and a unique, extra-terminal domain. They are highly conserved and form a distinct subfamily with similar hydrophobic binding pockets (5, 6).

Bromodomain protein 4 (BRD4) is the best studied member of the BET family. It is a nuclear protein that binds to acetylated histone 3 (H3) and histone 4 (H4) tails and plays an essential role in maintaining chromatin architecture (7). Through its direct interaction with the acetylated cyclin T1 subunit of the positive transcription elongation factor b, BRD4 furthermore controls transcription elongation via phosphorylation of RNA polymerase II by CDK9 (8–11). Genome-wide studies indicate that BRD4 binding correlates with gene expression and that besides promoter regions, intergenic and intragenic regions are also recognized (12). BRD4 also binds to acetylated RelA, thus stabilizing nuclear NF-κB and controlling the expression of downstream target genes (13, 14). In addition, the BRD4 extra-terminal domain interacts with several chromatin modifiers, including the histone methyltransferase NSD3 (15).

Elucidation of the function of histone acetylation mark readers was much supported by the recent discovery of specific small-molecule inhibitors (16). BET-selective inhibitors such as JQ1 and I-BET151 (17–19) helped to demonstrate the essential role of BRD4 in growth control. Several BRD4 target genes, including c-Myc, c-Fos, and cyclin D1, are involved in cell cycle control (20–23), and BRD4 binding is much enriched in super-enhancer regions of tumor-associated genes, such as c-Myc and FOSL2 (22). Consequently, BRD4 is implicated in several proliferative diseases. For example, chromosomal translocation leading to the expression of a fusion between BRD4 (or BRD3) and the nuclear protein in testis (NUT) is causative of a rare cancer form named NUT midline carcinoma (NMC) (24, 25). Patient-derived NMC tumors are inhibited by the BET inhibitor JQ1 in *in vivo* xenograft models, and the first clinical studies addressing this indication have already been initiated (17, 26). BRD4 also plays a crucial role in a number of hematological malignancies including acute myeloid lymphoma (19, 27), acute lymphoblastic leukemia (28), lymphoma (21), pediatric B-precursor acute lymphoblastic leukemia (28), and multiple myeloma (29). In line with this, clinical studies mainly addressing hematological tumors have recently been started. Furthermore, anti-proliferative effects of BET inhibition in solid tumors such as glioblastoma (30), neuroblastoma (31), lung cancer (32, 33), and melanoma (34) have been reported. Another pathology in which BRD4 is implicated is inflammation, as evidenced by the protective role of the BET inhibitor I-BET762 against endotoxic shock and sepsis (18). Finally, hijacking of BRD4 activity is essential for the life cycle of a number of viruses, including herpes and papilloma viruses (35). These pathogens take advantage of the retention of BRD4 to the host mitotic chromosomes for their propagation during cell division.

As mentioned, the interaction between BET bromodomains and acetyl-lysine is essential for cellular function. Bromodomains are composed of ∼110 amino acids that form a left-handed bundle of four α helices (αZ, αA, αB, αC) linked by the highly variable ZA and BC loop regions and constitute a deep, hydrophobic substrate binding pocket (36). Co-crystal structures of BET bromodomains and bound histone-derived peptides reveal that the acetyl-lysine side chain is anchored by a hydrogen bond formed with a conserved asparagine (*e.g.* Asn-140 in BRD4 BD1) located in the BC loop and also found in other bromodomains (37, 38). NMR spectroscopy of BRD4 BD2 associated to NF-κB-K310(ac) allowed the identification of key interacting amino acids including Asn-433, which makes a direct hydrogen bond with acetylated lysine (13). Additional amino acids in the ZA loop and in the αB and αC regions have been reported to be key for acetyl-lysine recognition (17). Several water molecules preventing further direct contacts are found at the bottom of the bromodomain pocket (39). X-ray structures solved in the presence of BET inhibitors such as JQ1 or I-BET762 show that these compounds effectively mimic the acetyl-lysine moiety (17, 18).

Although crystal structures can provide a static overview of the residues involved in interactions with substrates and small molecules, only a detailed mutational analysis of these residues can unravel their precise contributions to binding affinity. Up to now only a few such studies have been performed. The first reported BET mutants focused on the equivalent Tyr-139 and Tyr-432 or on Tyr-139 and Val-439 residues in BRD4 BD1 and BD2, respectively. These mutants have increased mobility and impaired interaction with acetylated chromatin in comparison to the wild-type form (40). Recently it was shown that mutating Asn-140 and Asn-433 in BRD4 BD1 and BD2, respectively, abolishes the binding to di-acetylated H4 peptides in a SPOT assay as well as in isothermal calorimetry, confirming the importance of the hydrogen bond formed by the highly conserved asparagine residue (4). Asn-140 and the neighboring Tyr-139 in BRD4 BD1 as well as the equivalent positions in BD2 are also important for the interaction with acetylated RelA (14). In the case of BRD2, surface plasmon resonance (SPR) reveals that additional BD1 residues including Tyr-113, Asn-156, and Asp-160 are essential for binding to a mono-acetylated H4 peptide (38). This was confirmed for Tyr-113 and its BD2 counterpart in living cells (41) and by immunoprecipitation (42). In murine BRDT, the Ile-114 mutant (43) and the triple mutant modified at positions Pro-50, Phe-51, and Val-55 (which correspond to Ile-112, Pro-48, Phe-49, and Val-53 in human BRDT) or at the equivalent positions in BD2 lose their binding to the H4 N-terminal tail (44). For BRD3, a detailed analysis of its interaction with acetylated GATA1 has been reported (45). Mutation of several hydrophobic residues has strong effects whereas, interestingly, mutation of the conserved Asn-116 residue, which forms the important hydrogen bond with the acetyl group of histone peptides, has little impact on binding, suggesting that, at least in this particular case, alternative recognition mechanisms are possible. Altogether, these examples indicate that disturbing individual interactions between BET bromodomains and their acetylated protein partners can have a significant impact on substrate binding, raising the question of whether these findings would be applicable to all members of the BET family and to interactions with small molecule inhibitors. The latter question can be relevant in the context of clinical use of BET inhibitors. For instance, following the approval of tyrosine kinase inhibitors, a number of resistance mechanisms originating from single point mutations of the targeted kinase have been identified in patients (46). Frequently, these mutations decrease the affinity of the drug for the kinase domain, whereas the catalytic activity is maintained. Furthermore, mutations affecting amino acids surrounding the binding site of the drug and reducing the availability of the target region toward the inhibitor without interfering with ATP binding have been described (47). Also, some mutations increase the affinity of the kinase for ATP, thus reducing the efficacy of the ATP-competitive inhibitors (48).

As the first BET inhibitors have just entered the clinic, no drug-induced resistance mutation has yet been described. Nonetheless, an early understanding of which bromodomain residues are likely to be involved in escape mechanisms from current BET small-molecule inhibitors may already guide the development of next-generation compounds.

The aim of this study was to investigate systematically with several complementary methods the structural determinants for the interaction of BRD4 with their natural ligands and inhibitors. To this end we evaluated the effect of mutations in the acetyl-lysine binding pocket of BRD4 BD1 on the recognition of various acetylated substrates and of the BET inhibitor JQ1. Furthermore, we analyzed in detail the contribution to the binding of amino acid residues flanking the acetyl-lysines of the H4 N-terminal tail. Our results should serve to advance the current understanding of the mechanisms underlying selective chromatin recognition by bromodomains and may help to guide the future design of more potent and specific BET inhibitors.

## EXPERIMENTAL PROCEDURES

### 

#### 

##### Plasmids

The His-tagged human BRD4 BD1 expression construct pNIC28-Bsa4 (construct ID BRD4A-c002) and BRD4 BD2 expression construct pNIC28-Bsa4 (construct ID BRD4A-c011) have been described (17). For the photobleaching experiments we used full-length human BRD4 cDNA (RefSeq NM_058243.2) cloned into the pcDNA6.2/N-EmGFP-DEST plasmid (Invitrogen) by the Gateway technology (Invitrogen) to create a chimeric green fluorescent protein (GFP)/wild-type BRD4 expression construct. For the cellular stabilization assays, the codon-optimized sequence of the wild-type and mutant versions of BRD4 BD1 (amino acids 44–166) flanked by an N-terminal His_6_ tag and a C-terminal FLAG tag and by 5′ and 3′ site-specific attachment sites for recombination using the Gateway technology (Invitrogen) were prepared by total DNA synthesis (GeneArt). The synthesized DNA fragments were cloned into the pTT5 (49) destination vector using a lambda site-specific recombination system (LR-recombination, Gateway Technology, Invitrogen). Plasmid DNA was prepared using the QIAprep Spin Miniprep kit (Qiagen).

##### Peptides

Peptides corresponding to wild-type and mutant histone H4 (1–25), to histone H3 (1–21), and to RelA (301–320) were synthesized and purified at Biosyntan and AnaSpec with or without a C-terminal biotin tag. Their purities (>90%) and identities were verified by liquid chromatography mass spectrometry (LC-MS).

##### Crystal Structures

The crystal structures of BRD4 in complex with the acetylated H4 K5(ac)K8(ac) peptide and JQ1 (PDB codes 3UVW and 3MXF, respectively) were downloaded from the RCSB protein data bank and analyzed with the Maestro software (Schrödinger Release 2013-2: Maestro, Version 9.5, Schrödinger, LLC, New York, 2013) and with PyMOL (PyMol Molecular Graphics System, Version 1.3, Schrodinger, LLC).

##### Site-directed Mutagenesis

Point mutations were introduced into the pNIC28-Bsa4 and pTT5 constructs using the QuikChange II XL Site-directed Mutagenesis kit (Agilent Technologies) following the manufacturer's instructions. Mutagenesis primers and their reverse sequence were synthesized by Eurofins MWG Operon and TIB MOLBIOL. The generated products were digested with DpnI for 1 h at 37 °C and transformed into XL10-Gold or XL1-Blue competent bacteria (Agilent Technologies). Plasmid DNA was purified from individual colonies as described above. The presence of the desired mutations was confirmed by DNA sequencing (SMB Services in Molecular Biology).

For the fluorescence recovery studies, mutant coding sequences were introduced into pcDNA6.2/N-EmGFP-BRD4 using the megaprimer PCR method (50) to amplify a BamHI/KpnI-flanked region (BD1) or KpnI/EcoRI-flanked region (BD2) of the wild-type expression plasmid using AccuPrime Pfx (Invitrogen). The expression plasmid and PCR products were digested with the appropriate restriction enzymes (New England Biolabs) followed by gel purification, dephosphorylation of the cut expression plasmid (Antarctic phosphatase, New England Biolabs), and ligation (T4 ligase, New England Biolabs) of the fragments to generate mutant GFP-tagged expression clones.

##### Protein Expression and Purification

BRD4 domains and mutants were expressed essentially as described (17) using One Shot BL21(DE3)pLysS bacteria stimulated with 0.5 mm isopropyl-β-d-thiogalactopyranoside (Sigma). Bacteria expressing His_6_-tagged proteins BRD4 BD1 or BD2 wild type were resuspended in 50 mm HEPES, pH 7.5 (Applichem), 500 mm NaCl (Sigma), 5% glycerol (Sigma), 5 mm imidazole (Sigma), 0.5 mm Tris(2-carboxyethyl)phosphine (Pierce), and EDTA-free Complete, and lysed using a Microfluidizer (Microfluidics). The proteins were then purified by affinity chromatography using nickel-nitrilotriacetic acid-agarose (Macherey-Nagel) or His-trap columns (GE Healthcare) followed by a size exclusion chromatography step using a Superdex S75 26/60 column (GE Healthcare). The mutated variants were purified by affinity chromatography using the nickel-nitrilotriacetic acid Fast Start Kit (Qiagen) following the instructions for purification under native conditions. A second purification step by size exclusion chromatography using a Superdex S75 16/60 column was then performed. The fractions were collected and monitored by SDS-polyacrylamide gel electrophoresis.

##### LC-MS

LC-MS analysis of purified proteins was performed in a nanoAcquity ultraperformance LC system coupled to a SYNAPT G2-S mass spectrometer with an electrospray ionization source (Waters). The protein samples were applied to Mass Prep C4, 2.1 × 5-mm trapping columns (Waters) using water containing 0.1% formic acid as mobile phase and then transferred using an acetonitrile gradient (20–80% in 6 min) in the presence of 0.1% formic acid and at a flow rate of 100 μl/min. Data were acquired over an *m*/*z* range of 450–2500 for 3.3 min.

##### Thermal Shift Assay (TSA)

In a typical TSA, 0.4 μg of BRD4 BD1 protein were mixed with 5× Sypro Orange (Molecular Probe) and adjusted to a final volume of 5 μl with 100 mm HEPES buffer, pH 7.5 (Applichem), and 150 mm NaCl (Sigma). For binding experiments, 1% DMSO (Sigma) or JQ1 in serial dilution (0.14 μm to 100 μm, 3-fold) was added to the mixture. To protect samples from evaporation, they were overlaid with 1 μl of silicone oil DC200 (Sigma). Melting curves were recorded using a ThermoFluor system (Johnson and Johnson Pharmaceutical Research and Development) after heating the samples from 25 up to 95 °C while measuring the fluorescence intensity of the dye with the excitation and emission filters set to 465 and 590 nm, respectively. For data analysis, melting curves (fluorescence intensity changes at increasing temperatures) were fitted using a first derivative method (51) to determine the melting point *T_m_* and the unfolding enthalpy Δ*H*_u_ using the evaluation software provided by the manufacturer of the instrument. A series of thermal melting curves was collected in the presence of varying inhibitor concentrations. The heat capacity of protein unfolding (ΔCp_u_) was assumed to be independent of the temperature and estimated from the size of the protein using the equation (52), ΔCp_u_ = −119 + 0.20(−907 + 93(#residues)). The standard enthalpy of binding (Δ*H*°_b_) was calculated as follows (53): ΔH°_b_ = ΔH°_u(DMSO)_ − ΔH°_u(JQ1 100 μm_). The difference of positive free energy of unfolding (ΔΔ*G*_u_) was calculated for each inhibitor concentration using ΔΔ*G*_u(JQ1,T)_ = Δ*H*_u(JQ1)_(1 − *T_m_*_(DMSO)_/*T_m_*_(JQ1)_) + ΔCp_u_[*T_m_*_(JQ1)_ − *T_m_*_(DMSO)_ + *T_m_*_(DMSO)_ ln(*T_m_*_(DMSO)_/*T_m_*_(JQ1)_)] (53). The extrapolated standard free energy change ΔΔ*G*°_u_ value in the function of the JQ1 concentration was fitted using the GraphPad Prism software and the following equation to obtain estimate *K_D_*° values (53): *Y* = −R*T_m_*_(DMSO)_ln{1 + [X-Pt-2 × *K_D_*° + sqrt((X + Pt + 2 *K_D_*°)^2 − (4Pt*X*))]/(2 *K_D_*°)}, where R is the gas constant, and Pt is BRD4 concentration.

Standard free energy of binding (Δ*G*°) and entropy (Δ*S*°) were calculated with the equations Δ*G*° = −RTln(1/*K_D_*°) and −*T*Δ*S*° = Δ*G*° × Δ*H*°_b_. To facilitate comparisons dΔG° was also calculated: dΔ*G*° = Δ*G*° (mt) − Δ*G*° (WT).

##### Time-resolved Fluorescence Resonance Energy Transfer (TR-FRET)

The TR-FRET binding assay used to measure the interaction of BRD4 with acetylated peptides was a modified version of a previously described protocol (18). The experiments were performed in 384-well black small volume microtiter plates (Greiner) in a buffer composed of 50 mm Hepes, pH 7.5 (Applichem), 50 mm NaCl (Sigma), 400 mm KF (Sigma), 0.5 mm CHAPS (Sigma), and 0.05% BSA (Sigma) in a final volume of 5–10 μl (*n* = 4). BRD4 (100 nm) protein domains were mixed with 200 nm concentrations of the biotin-labeled histone peptides and incubated for 30 min at room temperature. The protein-peptide complexes at equilibrium were then detected with 5 nm Eu^3+^ cryptate-conjugated streptavidin (CisBio) and 10 nm anti-His_6_-XL665 (Cisbio). To analyze the binding of mutated histone peptides at different ionic strengths, streptavidin Eu^3+^ chelate (W1024, PerkinElmer Life Sciences) was used instead of cryptate as fluorescence donor, and KF was replaced by NaCl at concentrations of 20, 100, and 500 mm. After 3 h of further incubation at 4 °C, the plates were measured in a PheraStar reader (BMG Labtech) using the homogeneous time-resolved fluorescence module (excitation, 337 nm with 10 flashes; emission, 620 and 665 nm). The 665-nm/620-nm ratios were converted to Delta F (Delta F (%) = (ratio sample − ratio background) × 100/(ratio background)) to normalize experiments performed at different days and with different readers.

For competition assays 50 nm BRD4 BD1 and 500 nm BD2 protein were preincubated with serial dilutions of the unlabeled H4 (1–25) K5(ac)K8(ac)K12(ac)K16(ac) tetra-acetylated peptide (15 nm to 250 μm, 2-fold) or with JQ1 (0.61 nm to 10 μm, 2-fold) for 30 min at room temperature. The plates were then processed as described above. IC_50_ values were calculated by plotting the log [competitor/inhibitor] *versus* mean normalized response data from at least two independent experiments measured in triplicate and fitting it to a four-parameter equation with variable Hill slope, Y = bottom + (top-bottom)/(1 + 10^(logIC_50_ −^ *^X^*^) × Hill slope)^), using the GraphPad Prism software. The IC_50_ values obtained under these experimental conditions can be expressed as *K_D_* (= *K_i_*) based on the Cheng-Prusoff equation (54): *K_i_* = IC_50_/(1 + [L]/*K_D_*). Free energy of binding Δ*G* and dΔ*G* were calculated as described above.

##### SPR

SPR interaction analyses were conducted on Biacore 2000 and T200 instruments (GE Healthcare) using standard buffers and protocols (Biacore™ Assay Handbook). For peptide binding assays, BRD4 BD1 and BD2 (50 μg/ml in 25 mm HEPES, pH 7) were immobilized on 1000M polycarboxylate hydrogel sensor chips (Xantec) at densities varying between 2000 and 3000 RU using standard amine coupling methods based on ethyl(dimethylaminopropyl)carbodiimide/*N*-hydroxysuccinimide chemistry at densities varying between 2000 and 3000 RU. The analyte was an unlabeled tetra-acetylated H4 peptide flowed over the surface by injecting serial, 2-fold dilutions (0.16–20 μm) in running buffer (HBS-P+) over all surfaces at a flow rate of 30 μl/min. The association phase was followed during 60 s, and the complexes were allowed to dissociate in buffer during 600 s before regenerating the surface with 1 m NaCl. The kinetic and steady-state data obtained were fitted to the Langmuir 1:1 and a single-site equilibrium binding equation (Biacore^TM^ Assay Handbook) using the BIA evaluation software (Biacore, GE Healthcare).

To evaluate the binding of the BRD4 BD1 mutants to histone tails in comparison to the wild type, a reverse set-up was used. Briefly, SA sensor chips (Biacore, GE Healthcare) were coated by injecting a 10 μm solution of the biotinylated tetra-acetylated H4 peptide used for TR-FRET assays at a flow rate of 10 μl/min until capture levels of 1100–1200 RU were achieved. To validate the surface, the wild-type protein was titrated on the chip at concentrations varying from 0.02 to 20 μm, and the affinity parameters for the interaction were calculated as described above. After confirming that the *K_D_* was in agreement with known values, all mutant and wild-type proteins were injected at a single concentration of 10 μm until equilibrium was established. Double referenced binding responses at equilibrium were used to calculate the surface activity or binding percentage: % binding = (RUeq/*R*max) × 100, where *R*max describes the binding capacity of the surface ((*M*_r_ analyte/*M*_r_ ligand × *RL*, where RL is the immobilization level of the ligand (H4 peptide in this case)).

##### Fluorescence Polarization (FP)

The affinity of the BRD4 variants for JQ1 (*tert*-butyl[(6*S*)-4-(4-chlorophenyl)-2,3,9-trimethyl-6H-thieno[3,2-f][1,2,4]triazolo[4,3-a][1,4]diazepin-6-yl]acetate)) was estimated with an FP binding assay using the tetramethylrhodamine (TAMRA)-labeled JQ1 derivative 5-{[5-({[(6S)-4-(4-chlorophenyl)-2,3,9-trimethyl-6H-thieno[3,2-f][1,2,4]triazolo [4,3-a][1,4]diazepin-6-yl]acetyl}amino)pentyl]carbamoyl}-2-[6-(dimethylamino)-3-(dimethyliminio)-3H-xanthen-9-yl]benzoate. To validate this fluorescent probe, its ability to displace the H4 peptide in the TR-FRET assay described above was assessed beforehand, and the *K_D_* was found to be comparable with that of JQ1 (data not shown). For *K_D_* determinations, 10 nm JQ1-TAMRA probe was incubated in 384-well small volume black microtiter plates with serial (1:2) dilutions of each BRD4 variant (0.61 nm to 10 μm) in a buffer containing 10 mm HEPES, pH 7.5 (Applichem), 150 mm NaCl (Sigma), 0.005% Tween 20 (Sigma), and 0.5 mm Tris(2-carboxyethyl)phosphine (Calbiochem). After 30 min of incubation at room temperature, the plates were measured in a Tecan M1000 plate reader using its FP module and excitation/emission wavelengths of 530 and 570 nm, respectively. For *K_D_* determinations the background-corrected polarization values were plotted against the concentrations of BRD4, and the data were fitted to a one-site binding equation: *Y* = *B*max × *X*/(*K_D_* + *X*), where *Y* is the specific binding, and *B*max is the maximal binding, using the GraphPad Prism software. Free energy of binding Δ*G* was calculated as described above.

##### Fluorescence Recovery After Photobleaching (FRAP)

FRAP studies were performed using a protocol modified from previous work (17, 55). In brief, U2OS cells were reverse-transfected (Lipofectamine 2000, Invitrogen) with mammalian expression constructs encoding chimeric GFP-BRD4 (wild type or mutants). Where required, 1 μm JQ1 was added 1 h before imaging, which was carried out 24 h after transfection. The FRAP and imaging system consisted of a Zeiss LSM 710 scanhead (Zeiss GmbH) coupled to an inverted Zeiss Axio Observer, Z1 microscope equipped with a high numerical aperture (N.A. 1.3) 40× oil immersion objective (Zeiss GmbH) and a heated chamber set at 37 °C. FRAP and GFP fluorescence imaging were carried out with an argon-ion laser (488 nm) and with a photomultiplier tube detector set to detect fluorescence between 500–550 nm. A 13.4-μm^2^ circular region (diameter of 70 pixels) of a GFP-positive nucleus was selected, and after 5 pre-scans, the region was bleached. A time-lapse series was then taken to record GFP recovery using 1% of the power used for bleaching with an interval time of ∼0.25 s. The average intensity at each imaging time point was measured for three regions of interest: the bleached region (It), the total cell nucleus (Tt), and a random region outside of the cell for background subtraction (BG). The image datasets were exported from the microscope control software (ZEN 2010) into Microsoft Excel 2010, and the relative fluorescence signal in the bleached region was calculated for each time point *t* with the equation (56) (*T*_prebleach_ − BG)(*I_t_* − BG)/(*T_t_* − BG)(*I*_prebleach_ - BG). The base line was normalized to zero, and the prebleach was normalized to 1. Normalized data were imported into GraphPad Prism 6.0, and half-times of recovery (*t*_½_) were calculated from individual single exponential curve fits for each cell and presented as the mean for each group. *p* values were calculated using an unpaired *t* test.

##### Cellular BRD4 BD1 Stabilization Assay

HEK293–6E cells (Yves Durocher, National Research Council Canada) were grown in suspension with gentle shaking in FreeStyle F17 medium (Invitrogen) containing 4 mm l-alanyl-l-glutamine (BioChrom AG) and 0.1% Pluronic (Invitrogen). For transfection with DNA, 10 ml of cell suspension at a density of 10^6^ cells/ml were prepared. A mixture of 100 ng of plasmid DNA, 10 μg of pBlue Script II (as carrier DNA), and 20 μg of polyethyleneimine (Polysciences) in 1 ml of medium was kept at room temperature for 10 min, added to the cell suspension, and incubated at 37 °C, 5% CO_2_ overnight.

Transfected cells were centrifuged at 800 × *g* for 5 min, resuspended in assay medium (DMEM high glucose (4.5 g/liter) (PAA), 5 ml of l-glutamine (Sigma), and further diluted to 5 × 10^5^ cells/ml. A volume of 10 μl of the cell suspension was dispensed into the wells of a 384-well microtiter plate (Greiner) prefilled with 50 nl of compound solution in 100% DMSO. Plates were incubated at 37 °C, 5% CO_2_ for 24 h. Cells were lysed, adding 10 μl of homogeneous time-resolved fluorescence conjugate/lysis buffer (Cisbio) containing 13.3 nm anti-His D2 (Cisbio) and 0.866 nm anti-FLAG Tb (Cisbio) to the wells. Plates were incubated at room temperature in the dark for 2 h. Time-resolved fluorescence was measured with a PHERAstar FS microplate reader (BMG Labtech) using the homogeneous time-resolved fluorescence module (excitation 337 nm, emission 1: 620 nm; emission 2: 665 nm). The ratio of the signals at 665-nm/620-nm is proportional to the amount of tagged BRD4 in the lysate. Dilution series of JQ1 (10 concentrations, 3-fold dilutions, quadruplicates) were prepared in 100% DMSO, and 50 nl were added into the test plate with a Hummingbird device (Zinsser). Dose-dependent stabilization by JQ1 was evaluated using the 4-parameter nonlinear regression algorithm (Y = bottom + (top-bottom)/(1 + 10^(logEC_50_ −^ *^X^*^) × Hill slope^) of the GraphPad Prism software (GraphPad Software).

## RESULTS

### 

#### 

##### Binding of BRD4 Bromodomains to Acetylated Peptides

To validate the experimental methods chosen for this study and to obtain first quantitative information on the interaction between the wild-type form of BRD4 bromodomains and acetylated peptides, we measured by TR-FRET the binding of purified BRD4 BD1 and BD2 to 14 peptides covering the H3 or H4 tails and containing one or several acetylated lysines. In addition, we tested the binding to one peptide derived from the acetylated region of RelA recently shown to interact with BRD4 (57).

**FIGURE 1.**
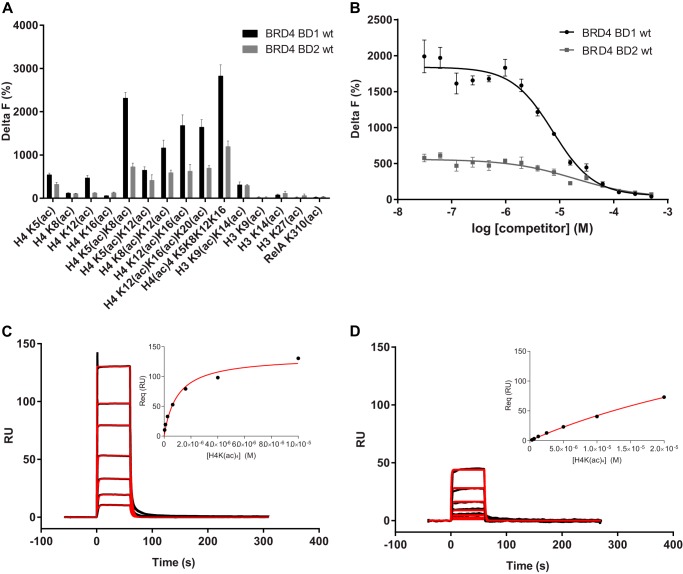
**Binding of BRD4 BD1 and BD2 to acetylated peptides.** *A*, TR-FRET quantification of BRD4 BD1 and BD2 (100 nm) binding to acetylated peptides derived from H3, H4 or RelA (200 nm each). *B*, determination of BRD4 BD1 and BD2 affinities to H4 K5(ac)K8(ac)K12(ac)K16(ac) using a TR-FRET homogeneous competition assay. 200 nm biotinylated H4 peptide and 50 nm BRD4 BD1 or 500 nm BRD4 BD2 were titrated with unlabeled H4 peptide at the concentrations indicated. Delta F values were plotted against the concentrations of unlabeled peptide (competitor). The fitting of data to the four-parameter equation described under “Experimental Procedures” (line) served to calculate the *K_D_* values indicated in Table 1. *C*, SPR sensorgrams of the interaction between BRD4 BD1 (immobilized on a 1000 m polycarboxylate chip) and histone H4 K5(ac)K8(ac)K12(ac)K16(ac). *D*, SPR sensorgrams of the interaction between BRD4 BD2 (immobilized on a 1000M polycarboxylate chip) and histone H4 K5(ac)K8(ac)K12(ac)K16(ac). The *insets* in *C* and *D* show SPR equilibrium resonance values Req *versus* [BRD4 BD1] and Req *versus* [BRD4 BD2] plots from which the *K_D_* values indicated in the text were calculated. *Red lines* represent the fit of the data to the Langmuir 1:1 and single-site equilibrium binding equations. All data are the mean values of at least two experiments with multiple replicates each.

The strongest binding signals were recorded for the interactions of BRD4 BD1 and BD2 with peptides bearing multiple acetylation sites, except for the H3K9(ac)K14(ac) and the H4K5(ac)K12(ac) peptides. On the other hand, only low binding to the mono-acetylated H3 and H4 peptides was observed (Fig. 1*A*). No significant binding to RelA was observed, in contrast to recent studies, possibly due to the fact that different constructs were used (14). In several, but not all cases, better signals were detected for BD1 than for BD2. Based on the strong signal measured, the tetra-acetylated H4 peptide was selected for a more detailed study. It was used as a displacement probe in a competitive binding assay with the aim of comparing in detail the affinities of the wild-type and mutant BRD4 bromodomains for this peptide. The exquisite sensitivity of the TR-FRET technology allowed the concentrations of BRD4 BD1 and BD2 and of the peptide to be kept far below the affinity constants previously reported for the interaction (4, 39, 58). Under these conditions, the non-biotinylated tetra-acetylated H4 peptide should quantitatively displace the fluorescent probe in a dose-dependent fashion and with an IC_50_ equivalent to the dissociation constant for the interaction (54). Using this experimental regime, dissociation constants of 4.8 and 30.6 μm were determined for BD1 and BD2, respectively (Fig. 1*B* and Table 1).

**TABLE 1:** **Affinity and energetics of BRD4 BD1 interaction with tetra-acetylated histone H4 peptide** Mean values of the affinity parameters derived from TR-FRET, SPR, and FRAP measurements (±S.D.) are listed. Values not shown could either not be interpreted (NI) in the TR-FRET competition experiments or were not determined (ND) in the FRAP studies.

Assay type	Readout	Parameter	BRD4 BD1 variant										
			WT	W81A	P82A	L94A	Y97F	Y139F	N140A	D144A	D145A	I146A	M149A
Competition (*in vitro*)	TR-FRET	*K_D_* (μm)	4.8 ± 0.4	52.2 ± 5.0	11.2 ± 0.7	39.8 ± 15	NI	8.5 ± 1.4	NI	6.9 ± 2.9	23.8 ± 3.3	17.1 ± 9.8	∼72.7 ± 10
		Δ*G* (kJ mol^−1^)	−30.4	−24.4	−28.3	−25.1		−28.9		−29.5	−26.4	−27.2	∼−23.6
Binding (*in vitro*)	SPR	Binding (%)*^a^*	100	10.8	29	9.7	1.6	27.8	0.2	36.5	18.7	23.8	34
Binding (in cells)	FRAP	*t*½ (s)	11.2 ± 5.8	3.3 ± 0.9	ND	ND	3.2 ± 0.9	ND	3.0*^b^* ± 0.8	ND	ND	ND	3.7 ± 1.0

*^a^* SPR surface activity normalized to wild type.

*^b^* Measured with N140F mutant.

To gain more insight into the binding mode and further validate the results described above, we additionally analyzed BRD4 BD1 and BD2 interactions with the H4 tetra-acetylated peptide using the SPR method. The binding sensorgrams obtained for both BRD4 bromodomains indicated fast equilibration kinetics for the interaction (Fig. 1, *C* and *D*), and the rates were often beyond the limits of what can be accurately measured by the reading instrument. Nevertheless, in the cases where the kinetic data could be reliably fitted to the Langmuir 1:1 model, the *K_D_*(kin) values derived from the kinetic parameters (*k*_on_ and *k*_off_, *K_D_*(kin) = *k*_off_/*k*_on_) were similar to the equilibrium affinities (data not shown). Plotting the steady-state responses at different analyte concentrations (*insets* in Fig. 1, *C* and *D*) yielded either full or partial binding saturation curves from which *K_D_*(eq) values of 4.9 and 32.5 μm were estimated for BD1 and BD2, respectively. These values were in excellent agreement with the affinity constants measured in the TR-FRET competition assay.

##### Influence of Amino Acids Neighboring Acetyl-lysines on H4 Recognition by BRD4 BD1

**FIGURE 2.**
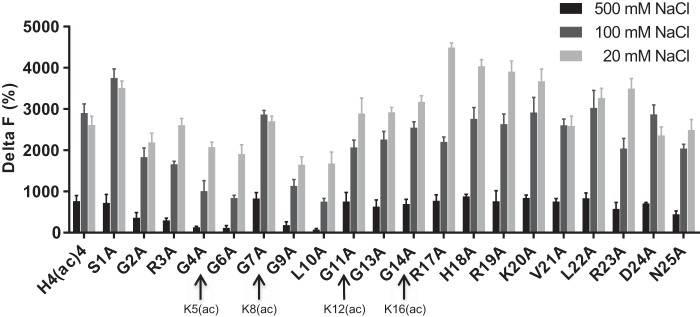
**Alanine scanning of the tetra-acetylated H4 tail.** The *bar chart* shows the average blank-subtracted TR-FRET Delta F (%) values corresponding to the interaction of BRD4 BD1 (100 nm) at three NaCl concentrations with tetra-acetylated histone H4 peptides (200 nm each) modified as indicated. The data represent the mean values with multiple replicates each.

The TR-FRET assay described above was used to analyze in detail the binding of BRD4 BD1 to tetra-acetylated H4 mutated at different positions and in the presence of different salt concentrations (Fig. 2). Altogether, we found that the interaction of all H4 peptides with BRD4 BD1 was strongly reduced at 500 mm NaCl concentration, compared with 20 and 100 mm. To determine if the amino acids of the H4 peptide located around the acetyl-lysine marks that do not directly interact with the bromodomain pocket nevertheless had an impact on the interaction, these residues were individually mutated to alanine, and the corresponding peptides were evaluated for BRD4 BD1 binding. Changing the residues that flank acetylated Lys-5 and Lys-K8 to alanine led to significantly weaker BRD4 BD1 binding, especially at high ionic strengths, with the notable exception of the Gly-7 position (Fig. 2). In contrast, mutations at positions Arg-17, His-18, Arg-19, or Lys-20 or at position Ser-1 slightly strengthened the binding of BRD4 BD1 to the acetylated H4 peptide at low salt concentrations.

##### Specific Contributions of Selected Amino Acids of the BRD4 Bromodomain Pocket to Binding Affinity

**FIGURE 3.**
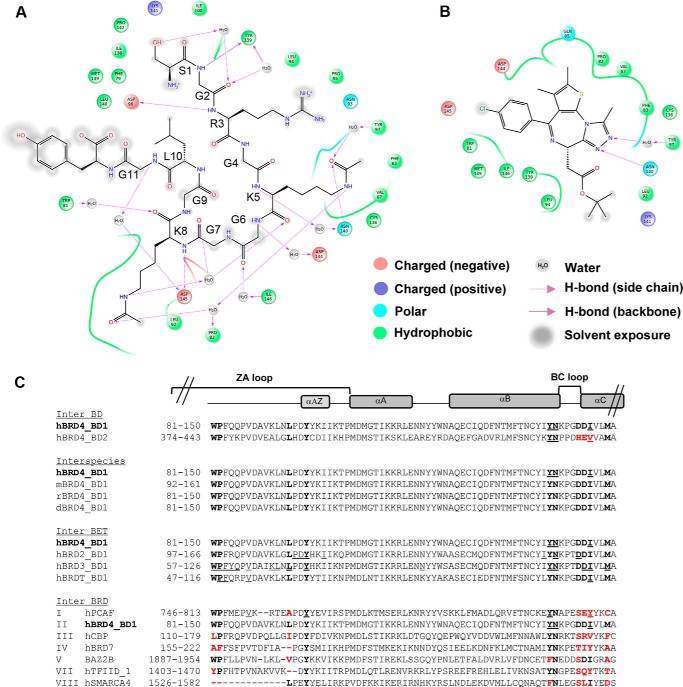
**Specific contacts of BRD4 with substrates and the small molecule JQ1 and conservation of key interacting residues within the bromodomain family.** *A*, two-dimensional representation of BRD4 BD1 contacts with K5(ac)K8(ac) H4 region as seen in the three-dimensional structure of the complex (PDB code 3UVW). *B*, two-dimensional representation of BRD4 BD1 interactions with JQ1 as seen in the three-dimensional structure of the complex (PDB code 3MFX). For *A* and *B*, properties of the interacting residues and nature of the interactions are indicated in the legend. *C*, alignment of selected bromodomain protein sequences. *Bold* characters indicate the residues that were mutated in the BRD4 BD1 sequence. Equivalent positions in other bromodomains are also in *bold* and highlighted in *red* when they are not conserved. Residues analyzed in previous works and mentioned in the text are *underlined. Roman* figures denote the different bromodomain subfamilies. No member of family VI is shown due to the low sequence conservation in the compared region. Secondary structure elements (α-helices) of human BRD4 are displayed *above the sequence alignment. h*, human; *m*, mouse; *r*, rat; *d*, *Drosophila*.

We next examined the individual interactions of BRD4 BD1 with H4 K5(ac)K8(ac) and with JQ1 in the available co-crystal structures (4, 17) in light of the conservation of the residues involved across the bromodomain family. This allowed selection of 10 amino acids whose mutations are likely to lead to measurable changes in binding affinity (Fig. 3). 4 of the 10 residues chosen are located in the ZA loop, namely the hydrophobic Trp-81 and Pro-82, which belong to the WPF shelf, and Leu-94 and Tyr-97, which face the binding pocket. Furthermore, we chose two residues in the αB helix, Asn-140, which forms the key hydrogen bond interaction but whose mutation in BRD3 intriguingly does not significantly affect binding to GATA1, and Tyr-139. Finally we selected the positively charged Asp-144 in the BC loop and Asp-145 and Ile-146 in the αC helix, which enclose the hydrophobic shelf, and Met-149, which may affect the ZA channel and the hydrophobic shelf, although not directly part of the pocket. These 10 amino acids are entirely conserved across BD1 in the BET family and play distinct roles in the interaction with histone H4 and JQ1 (Fig. 3, *A* and *B*). They are also found in BD2, with the exception of Asp-144, Asp-145, and Ile-146, which are substituted for histidine, glutamic acid, and valine, respectively (Fig. 3*C*). These residues were individually mutated to alanine, except Tyr-97 and Tyr-139, which were mutated to phenylalanine to keep the same overall conformation while eliminating the hydroxyl group that may undergo phosphorylation or be involved in hydrogen bonding.

Mutants of BRD4 BD1 were expressed and purified, and their expected mass and purity was confirmed by LC-MS analysis and SDS-PAGE, respectively (not shown). Differential scanning fluorimetry/TSA melting curves of mutated and wild-type BRD4 BD1 domains were comparable, indicating correct folding (not shown).

**FIGURE 4.**
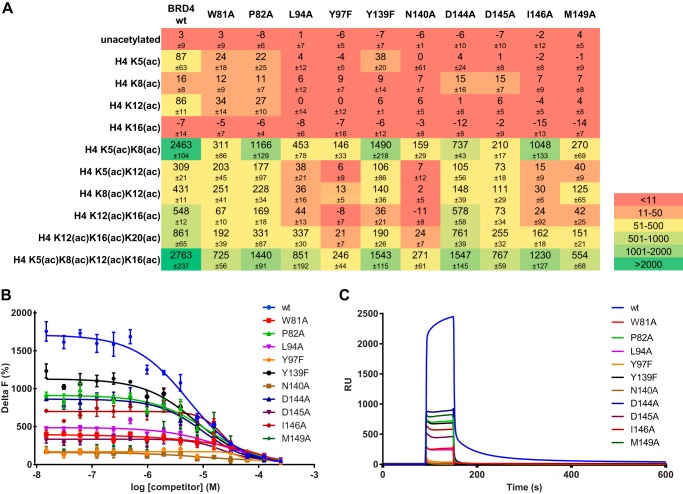
**Binding of BRD4 BD1 mutants to acetylated peptides.** *A*, TR-FRET delta F (%) quantification (±S.D.) of BRD4 BD1 (wild-type and mutants, 50 nm each) binding to different acetylated H4 histone tails (200 nm each). *B*, determination of BRD4 BD1 affinity to H4 K5(ac)K8(ac)K12(ac)K16(ac) with a TR-FRET homogeneous competition assay. 200 nm biotinylated H4 peptide and 50 nm protein were titrated with unlabeled H4 peptide at the concentrations indicated. Delta F values were plotted against the concentrations of unlabeled peptide (competitor). The fitting of the normalized data (not shown) to the four-parameter equation described under “Experimental Procedures” (*colored lines*) served to calculate the *K_D_* values indicated in Table 1. All data represent the mean values of at least two experiments with multiple replicates each. *C*, SPR sensorgrams of the interaction of BRD4 BD1 (wild-type and mutants, 10 μm each) with a biotinylated H4 K5(ac)K8(ac)K12(ac)K16(ac) peptide captured on a Biacore SA chip.

Binding of BRD4 BD1 mutants to H4 peptides was analyzed by TR-FRET as previously done for wild-type BD1 and BD2. Altogether, a reduction of the TR-FRET signals was observed for all mutants compared with the wild type (Fig. 4*A*). As for the wild-type BD1, the strongest binding was generally observed for the tetra-acetylated H4 peptide and the di-acetylated H4 peptide harboring acetylated Lys-5 and Lys-8. Conversely, very weak or no binding was seen for all mono-acetylated H4 peptides. Among the mutant proteins, P82A, Y139F, D144A, and I146A displayed the highest binding signals for the tetra-acetylated and most di-acetylated peptides. W81A, L94A, D145A, and M149A had significantly reduced binding activity, whereas Y97F and N140A showed the weakest interaction.

To confirm that the reduced TR-FRET signals at single concentrations of protein and peptide were an indicator of affinity decrease, we used the previously established TR-FRET competition assay for a precise quantification of the *K_D_* values. As expected, the P82A, Y139A, D144A, and I146A mutations had only a limited impact on the affinities in comparison to the wild-type protein. The affinities of D145A and L94A were more markedly reduced (5- and 8-fold, respectively), whereas for the W81A and M149A mutants the affinities were <10-fold that of the wild-type form (Fig. 4*B* and Table 1). The affinities of Y97F and N140A could not be quantified reliably due to the small dynamic window of the competition assay for these mutants.

The above-described results were further confirmed by SPR analysis of the interaction of immobilized H4 K5(ac)K8(ac)K12(ac)K16(ac) peptide with the purified BRD4 BD1 forms (Fig. 4*C*). We first validated the SPR surface by performing titrations of the wild-type BRD4 BD1 protein followed by steady state analysis of the sensorgrams (data not shown). The *K_D_* value of 2.4 μm calculated from this experiment was consistent with the values obtained in previously described assays. The RU and % surface activity (=% binding) values obtained from the injection of a single concentration (near *K_D_*) of wild-type or mutated bromodomain were, therefore, considered to be a *bona fide* estimate of the affinity of the protein for the immobilized peptide. In these experiments all mutants showed a reduced interaction compared with the wild-type form. The effects were most pronounced for W81A, L94A, Y97F, and N140A for which the remaining binding was roughly 10% or less (Table 1).

##### Impact of BRD4 BD1 Mutations on Nuclear Mobility

**FIGURE 5.**
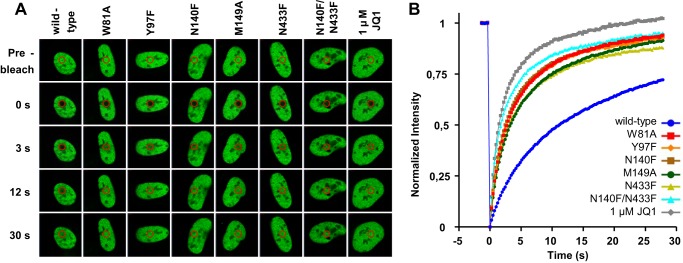
**Effect of bromodomain mutation on BRD4 dissociation from chromatin by FRAP evaluation.** *A*, nuclei of U2OS cells transfected with GFP-tagged wild-type BRD4, bromodomain mutants or with wild-type BRD4 and treated with JQ1. The bleached area is indicated by a *red circle. B*, time dependence of fluorescent recovery in the bleached area for wild-type, mutant, or treated cells. Curves represent the means at each time point of at least 12 cells in each group.

To evaluate the effect of the mutations in a more physiological context, we performed FRAP experiments to assess the intracellular mobility of BRD4 BD1 mutants. Because this technique only allows detection of major differences in behavior, we focused on the mutants we found to be the most impaired in their recognition of acetylated histone H4 peptides in biochemical assays, namely those with mutated Trp-81, Tyr-97, Asn-140, and Met-149. We transfected human osteosarcoma U2OS cells with full-length, GFP-tagged BRD4 individually mutated to alanine or phenylalanine at these four BD1 locations as well as with BRD4 BD2 mutated at Asn-433 (which corresponds to Asn-140 in BD1) and with a double Asn-140/Asn-433 mutant. Successfully transfected cells were subjected to FRAP analysis, and the half-recovery time of wild-type and mutant BRD4 bromodomains were determined. The Asn-140 and Asn-433 residues were modified to either alanine or phenylalanine residues with essentially the same results. As the transfection efficiency was better for the phenylalanine mutants, only these data are shown. Cells transfected with GFP-BRD4 wild type with or without treatment of the BET inhibitor JQ1 were used as references (Fig. 5*A*). All BRD4 BD1 and BD2 mutants showed a quicker recovery of fluorescence intensity than the wild-type protein in the cell nuclei, indicative of displaced and freely diffusing proteins. For all mutants, the enhanced half-fluorescence recovery was comparable to that seen for BRD4 wild-type in JQ1-treated cells (Fig. 5*B* and Table 1).

##### Affinity Determinants for BRD4 Recognition by the Small Molecule Inhibitor JQ1

**FIGURE 6.**
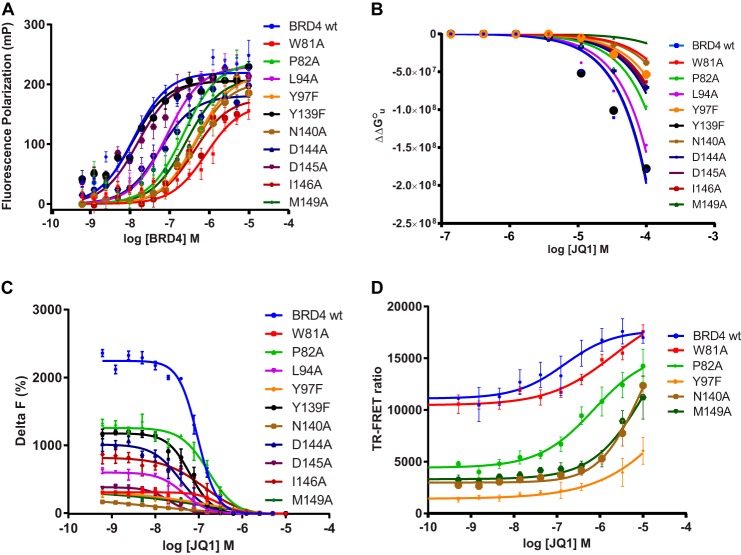
**Determination of BRD4 BD1 wild-type and mutant affinities for the small molecule inhibitor JQ1 *in vitro* and in cells.** *A*, FP analysis of BRD4 BD1 wild-type and mutant binding to TAMRA-labeled JQ1. The TAMRA-labeled JQ1 tracer (10 nm) was titrated with increasing concentrations of protein as indicated, and blank-subtracted fluorescence polarization values were plotted *versus* protein concentration. The *lines* show the fit of the data to a single-site equilibrium binding equation described under “Experimental Procedures.” The *K_D_* values obtained from these fits are indicated in Table 2. *B*, TSA analysis of BRD4 BD1 wild-type and mutant binding to JQ1. The compound-induced changes in free energy of unfolding (ΔΔ*G*°_u_) were plotted *versus* JQ1 concentration, and the data were fitted to the equation described under “Experimental Procedures” (*colored lines*). These fits served to calculate the *K_D_*° values shown in Table 2. *C*, TR-FRET competition assay. 200 nm biotinylated H4 peptide and 50 nm wild-type or mutant proteins were titrated with JQ1 at the concentrations indicated in the graph. Plots of the Delta F data *versus* JQ1 concentration were fitted to the four-parameter equation described under “Experimental Procedures” (*colored lines*) to calculate the *K_D_* values indicated in Table 2. *D*, cellular stabilization of BRD4 wild-type and mutants by JQ1. HEK293 cells expressing wild-type and mutant variants of BRD4 BD1 were incubated with JQ1 and tested following the protocol given under “Experimental Procedures.” Fitting the plots of TR-FRET ratio at increasing JQ1 concentrations was used to calculate the EC_50_ values for protein stabilization.

Although JQ1 efficiently mimics the acetyl-lysine group and occupies the same space in the BRD4 BD1 pocket (17), it does not make exactly the same interactions with the protein as do the acetylated histones (Fig. 3, *A* and *B*). We, therefore, decided to compare in detail the respective interactions of JQ1 and the tetra-acetylated H4 peptide with the different BD1 point mutants we generated. An important point we wished to investigate was whether the reduction of BRD4 BD1 mutant interaction with histone peptides translated into a quantitative loss of inhibitor binding. To this end a TAMRA-labeled JQ1 derivative was titrated with increasing concentrations of the mutants, and FP of the probe was used as indicator for complex formation (Fig. 6*A*). The results summarized in Table 2 indicate that JQ1 binding to the Y139F and D145A mutants was not significantly different compared with wild-type BRD4 BD1. The strongest effects were observed for P82A, Y97F, N140A, I146A, and M149A and especially for the W81A mutant.

**TABLE 2:** **Affinity and stabilization thermodynamics of BRD4 BD1 interaction with JQ1** Mean values of the affinity and thermodynamics parameters derived from FP, TSA, and TR-FRET measurements (±S.D.) are listed. Values not shown either could not be interpreted (NI) in the TR-FRET competition experiments or were not determined (ND) in the protein stabilization studies.

Assay type	Readout	Parameter	BRD4 BD1 variant										
			WT	W81A	P82A	L94A	Y97F	Y139F	N140A	D144A	D145A	I146A	M149A
Binding (*in vitro*)	FP	*K_D_* (nm)	14.5 ± 3.3	834.7 ± 281.5	229.3 ± 59.7	85.3 ± 14.8	474.8± 73.8	11.4 ± 2.2	447.8 ± 70.6	56.6 ± 11.3	15.8 ± 3.7	414.8 ± 100.7	267,8 ± 77.1
		Δ*G* (kJ mol^−1^)	−44.7	−34.7	−37.9	−40.4	−36.1	−45.3	−36.2	−41.4	−44.5	−36.4	−37.5
	TSA	Δ*T_m_**^a^* (°C)	9.6 ± 0.6	2.8 ± 0.4	5.1 ± 0.2	6.9 ± 0.4	3.1 ± 0.6	9.8 ± 0.3	3.3 ± 0.4	9.5 ± 0.6	9.2 ± 0.6	6.1 ± 0.3	3.2 ± 0.3
		*K_D_*^o^ (nm)	1.0	13.7	3.64	2.14	10.5	1.0	8.6	1.0	1.1	2.4	9.7
		Δ*G*^o^ (kJ mol^−1^)	−51.3	−44.9	−48.2	−49.5	−45.6	−51.5	−46.0	−51.3	−51.2	−49.2	−45.7
		Δ*H*^o^_b_ (kJ mol^−1^)	−13.8	−9.2	−17.4	−17.7	−15.5	−18.7	−11.9	−9.9	−6.5	−8.8	−0.5
		−*T*Δ*S*^o^ (kJ mol^−1^)	−37.5	−35.7	−30.8	−31.8	−30.1	−32.8	−34.1	−41.4	−44.7	−40.4	−45.3
Competition	TR-FRET	*K_D_* (nm)	105.7 ± 13.9	NI	174.1 ± 13.9	42.8 ± 2.3	NI	61.3 ± 16.5	NI	44.0 ± 11.4	16.8 ± 4.6	112.0 ± 23.5	NI
		Δ*G* (kJ mol^−1^)	−39.4		−38.2	−41.6		−40.8		−41.6	−43.9	−39.3	
Binding (in cells)	Protein stabilization	EC_50_ (μm)	0.1	2.0	0.7	ND	>10.0	ND	8.4	ND	ND	ND	>10.0

*^a^* Measured with 100 μm JQ1.

To test whether the results obtained using a JQ1 derivative recapitulated the interactions of the parent compound with BRD4, we analyzed the effects of JQ1 on unfolding thermodynamics of the BRD4 BD1 mutants using TSA. To this end we recorded melting curves of the wild-type and of each mutant at increasing ligand concentrations. As previously reported (17), JQ1 binding resulted in thermal stabilization of BRD4 BD1, and raising ligand concentrations increased the protein thermal stability (not shown). Analysis of the melting points of the mutants in the presence of 100 μm JQ1 revealed that the W81A, Y97F, N140A, and M149A forms were the least stabilized by JQ1, whereas the mutants P82A, L94A, and I146A showed a moderate stabilization profile (Table 2). Conversely, JQ1 stabilized the Y139F, D144A, and D145A mutants as strongly as wild-type BD1. The Δ*T_m_* values at 100 μm JQ1 were in good agreement with the affinities of the mutants for JQ1 in the FP assay (Table 2). Encouraged by these results, we used the Δ*T_m_* and Δ*H*_u_ parameters obtained from the experiments to estimate the binding enthalpy Δ*H*°_b_ and evaluate the impact of JQ1 on the protein stability change ΔΔ*G*°_u_ (Fig. 6*B*). Using the appropriate equations and making assumptions discussed elsewhere (52, 53, 59), we also derived *K_D_*° values for the interaction of JQ1 with the different mutants. The results of these studies are summarized in Table 2. The calculated standard binding enthalpy Δ*H*°_b_ of BRD4 BD1 wild type to JQ1 of −13.8 kJ mol^−1^ was in the same range as the −8.42 kcal mol^−1^ Δ*H*_obs_ previously measured by isothermal calorimetry (17). Loss of stabilization by JQ1 translated into loss of relative free energy of binding Δ*G*°, which, however, remained negative along with the binding enthalpy Δ*H*°_b_ and entropy changes *T*Δ*S*° (Table 2). As anticipated from the Δ*T_m_* values, the thermodynamic data obtained from the TSA experiments supported the affinity ranking of the mutants obtained using the FP assay.

The effects of BD1 mutations on JQ1 binding are also expected to influence the inhibitory activity of the compound. We, therefore, determined the interaction of JQ1 with each purified mutant in the TR-FRET competition assay using biotinylated H4 tetra-acetylated peptide as tracer (Fig. 6*C*). We calculated the *K_D_* of JQ1 for those mutants with sufficient affinity to allow an appropriate assay window. W81A, Y97F, N140A, and M149A were, therefore, excluded from this analysis. The results are summarized in Table 2. Surprisingly, the most dramatic effect was seen with D145A, where a 6-fold increase of inhibitory activity was observed.

##### Impact of BRD4 BD1 Mutations on JQ1-Mediated Protein Stability in Cells

To assess the relevance of our findings with JQ1 in a cellular context, we built on the fact that ligand-induced stabilization of proteins has recently been shown to take place in living cells (60) and hypothesized that small molecule inhibitors of BRD4 bromodomains can increase the half-life of the targeted protein in cells. To this end we developed a novel assay system that detects changes in BRD4 BD1 protein levels in cell lysates by TR-FRET. As shown in Fig. 6*D*, incubation of cells expressing BRD4 BD1 with JQ1 led to a dose-dependent and saturable increase of the TR-FRET signals, indicative of increased protein levels. The EC_50_ value measured for stabilization of wild-type BRD4 was 0.1 μm, in concordance with the IC_50_ determined in the biochemical TR-FRET competition experiment (Table 2). In line with the biochemical data, all mutated BRD4 variants that were least recognized by JQ1, namely Trp-81, Pro-82, Tyr-97, Asn-140, and Met-149, showed a much higher EC_50_ in the cellular stabilization assay (from 7-fold to >100-fold), indicative of a reduced recognition by JQ1 inside cells. The effects were most pronounced for the N140A and especially the M149A mutants.

## DISCUSSION

The BET BRD4 protein plays a major role in cancer development as a reader of acetylated histones, and first clinical studies with inhibitors targeting BET bromodomains have already been initiated. A detailed understanding of the bromodomain residues engaged in acetylated lysine and inhibitor binding will be essential for the identification of compounds with an improved pharmacological profile. Mutations in epigenome regulators, including histone mark readers, have already been described in several cancer types, and their efficient targeting may be the scope of next generation inhibitors (61).

Using different biophysical and biochemical methods, we first compared the binding profiles of BRD4 BD1 and BD2 to different acetylated H4 and H3 peptides and thereafter analyzed the contribution of individual BD1 amino acids. The results of the binding measurements allowed us to identify the tetra-acetylated peptide H4 K5(ac)K8(ac)K12(ac)K16(ac) and the di-acetylated peptide H4 K5(ac)K8(ac) as the best binding partners of BRD4 BD1. Furthermore, the fast kinetics of the interactions between BRD4 BD1 or BD2 and the tetra-acetylated H4 were shown for the first time. BRD4 BD1 had a >6-fold higher affinity for the tetra-acetylated H4 peptide than BD2, with affinity constant values comparable to those previously reported using isothermal calorimetry isothermal calorimetry (4). Altogether, these results strongly suggest that the first bromodomain is the main player in BRD4 recognition of acetylated histones. We also observed that binding of BRD4 to tetra-acetylated H4 was strongly impaired at high salt concentrations, raising the question of whether transient local changes of ionic strength in the nucleus may influence the binding of BRD4 to chromatin. A weaker interaction was observed for a H3-derived di-acetylated peptide, in line with previous data generated with peptide arrays. These studies analyzed the recognition of numerous acetylated histone-derived peptides by different BET and non-BET bromodomains using SPOT arrays. They revealed important differences in the recognition pattern, including individual binding profiles for the BET BD1 and BD2 regions (4). The limited binding seen for mono-acetylated peptides suggests that synergistic effects between different acetylation marks are needed for recognition. Indeed, this is a general feature found for most bromodomains, as previously shown by peptide array and isothermal calorimetry data (4). Also, structural studies performed with BRDT have revealed that two acetylation marks are recognized by a single bromodomain, thus mediating cooperative effects (43). The distance between acetylation marks was also noted to be important. A six-amino acid space between H4 acetylated lysines reduced the binding of BRD4 BD1 compared with a two- or three-residue spacing. Indeed the two closely spaced acetylation marks of the H4K5(ac)K8(ac) have been shown to both fit into BRD4 BD1 by cocrystallization (4).

We furthermore found that the nature of the amino acids located between modified lysines had an impact on the interaction of the tetra-acetylated H4 peptide with BRD4 BD1. Multiple glycine residues are present in the H4 tail between Lys-5 and Lys-16, suggesting flexibility of this region to be important for recognition by reader proteins. First experiments performed with a di-acetylated peptide had already shown that Gly-6, but not Gly-7, needed to be conserved to maintain strong binding by BRD4 BD1 (4). We confirmed the role of Gly-6 in the tetra-acetylated H4 peptide and additionally showed that Gly-4 and Gly-9 also had an impact on accessibility and recognition by BRD4 BD1. The importance of glycine residues near histone marks has recently been reported in childhood tumors where a mutation in H3.3 leading to a glycine to arginine or valine exchange modified the neighboring H3K36me3 profile (62). Interestingly, we also found the loss of binding to be dependent on the salt concentration. Given the position outside of the BRD4 binding pocket and the solvent exposure of the residues, this strongly suggests that the solvent interferes with the H4 conformation needed for optimal binding to BRD4. Leu-10 makes hydrophobic interactions outside of the binding pocket, which may explain the impact of the mutation on binding. Surprisingly, mutating the polar and charged Arg-17, His-18, Arg-19, or Lys-20 to alanine slightly increased the binding of BRD4 BD1 to the acetylated H4 peptide at low ionic strength conditions. These residues do not engage in the binding pocket, suggesting an indirect underlying cause such as higher solubility of the mutated histone peptide in low salt concentrations compared with the non-mutated peptide form.

As exemplified in the case of tyrosine kinase inhibitors, point mutations in protein regions targeted by drugs are a frequent escape mechanism observed in treated cancer patients. No such example exists for BRD4, but clinical studies are still in their early phase. We, therefore, studied in detail the role of individual BRD4 bromodomain residues in the interaction with acetylated histones and small molecule inhibitors. The crystal structures of BRD4 BD1 bound to the acetylated H4 peptide or to different inhibitors show the compounds to mimic acetyl-lysine but also to engage in somewhat different interactions. Based on these observations we selected 10 amino acids belonging to the binding pocket for site-directed mutagenesis and thorough analysis of the corresponding mutated BRD BD1 region.

**FIGURE 7.**
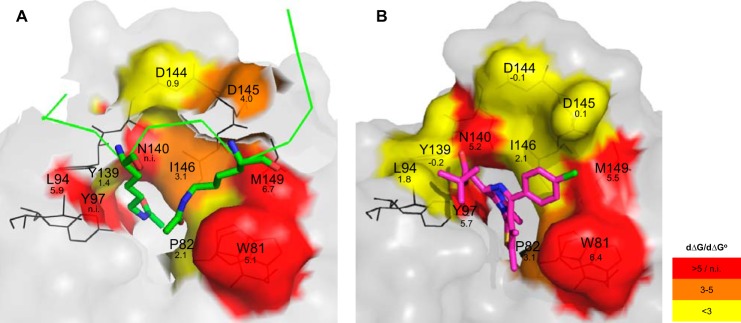
**Three-dimensional affinity map of BRD4 interactions with H4 tail and JQ1.** Residues investigated in this study are highlighted in the crystal structures of BRD4 complexed with H4K5(ac)K8(ac) (*A*) and JQ1 (*B*). The colors represent the loss of absolute free energy of binding (dΔ*G*) associated with the individual mutations. dΔG values were calculated by subtracting free energy of binding of the wild-type from the mutants. Non-mutated BRD4 regions are shown in *gray*, the H4K5(ac)K8(ac) peptide is shown in *green*, and JQ1 is in *magenta*. The dΔG values were calculated from the TR-FRET experiment (*panel A*) and the dΔG° values from the TSA experiment (*panel B*). *n.i.*, not interpreted.

The affinity of these mutants for H4 and JQ1 was compared with the wild-type form using the free energy of binding calculated from the competitive TR-FRET and TSA experiments. Altogether, the results allowed us to draw a three-dimensional affinity map of the BRD4 pocket engaged in interactions with histone H4 (Fig. 7*A*) or with JQ1 (Fig. 7*B*). The loss of absolute free energy of binding of each mutant compared with wild-type BD4 BD1 was color-coded depending on the difference measured. From these results, three different groups of mutations can be identified.

We first focused on mutants where important losses of histone and JQ1 binding were noted. The prime example is Asn-140, for which a strong reduction of binding properties was expected based on previous studies due to the formation of an essential hydrogen bond with the carbonyl group of the acetyl mark and with the 3-methyl-1,2,4-triazole ring of JQ1 (63, 64). Indeed, the Asn-140 position has already been shown to be essential for the reading function of many but not all bromodomains (4). For instance, the corresponding mutation in BRD3 BD1 has no impact on binding to GATA1 (45), suggesting that the interaction between a bromodomain and its acetylated partner may in some instances follow different rules. The Tyr-97 mutant also had severely impaired binding properties. Cocrystal structures indicate that Tyr-97 interacts indirectly by hydrogen bonding through a water molecule with the acetylated lysine (39) and also with JQ1 (17). The effects we observed are in line with comparable mutations in BRD2 and BRD3 showing Tyr-97 to be critical for binding to mono-acetylated H4 (38) and to GATA1 (45), respectively. Interestingly, molecular dynamic simulations (65) showed both Asn-140 and Tyr-97 as well as Tyr-139 to be located in a flexible region (see also Fig. 7*A*) allowing rotation between an inward and an outward position, which may affect recognition of the acetyl-lysine binding site. Two other residues were essential for peptide and inhibitor binding, namely the hydrophobic amino acids Trp-81 and Met-149. Both positions are known to be essential in BRD3 BD1 for its interaction with the GATA1 peptide (45). Interestingly, an alanine residue is found at the Trp-81 position in the BRD7 bromodomain, which has only low binding affinity for acetylated peptides (66). Trp-81 together with Pro-82 and Phe-83 form the hydrophobic WPF shelf (6, 67). In our study the hydrophobic interaction between JQ1 and Trp-81 was as important as the hydrogen bonds formed with Tyr-97 and Asn-140. The effects were less pronounced for the Met-149 mutation but still very marked. Importantly Trp-81 and Met-149 together with Ile-146 form the hallmark signature of the BET bromodomain group and are predictive of high drug-ability (6). In the BET family, the position corresponding to Met-149 is conserved, whereas in the BAZ family it is replaced by the small amino acids alanine or cysteine, which allow the tryptophan to move close to residue 149, thereby removing the ZA channel and hydrophobic shelf (6). Altogether these data show that Trp-81, Tyr-97, Asn-140, and Met-149 are key amino acids for histone and JQ1 binding, which is why we additionally analyzed them in a cellular context. Using FRAP, we observed an impaired chromatin recognition for all of the corresponding mutants. In a cellular stabilization assay performed in the presence of JQ1, reduced binding was also observed. Altogether these data demonstrate the bromodomain pocket of these mutants to lack essential residues for acetyl-lysine and inhibitor binding. These four residues can, therefore, be grouped based on their key role in the interaction with both acetylated histone H4 and JQ1.

A second group with a mixed profile was identified. It includes residues Pro-82, Ile-146, Leu-94, and Asp-145. The P82A mutation had little effect on H4 peptide binding but led to affinity loss toward JQ1 (Fig. 7). These results show that the interactions of Pro-82 with H4 that include two indirect hydrogen bonds with the acetylation marks are rather weak, whereas the interactions with JQ1 are strong due to hydrophobic forces engaged with the thiophene group of the compound. The I146A mutation showed a strong loss of affinity for H4, whereas binding to JQ1 was only partially affected. Interestingly, the nature of this gatekeeper residue varies outside of the BET family, suggesting this position to be essential for the diversity of bromodomain functions. In BRDT, introducing a large, bulky residue at this position entirely suppresses reorganization of hyper-acetylated chromatin (43). The L94A mutation led to a strong loss of affinity to the histone H4 tail comparable to that seen for members of the first group, whereas binding to JQ1 was only weakly affected. Concerning Asp-145, the affinity loss calculated for the mutant indicates that this residue forms medium strength hydrogen bonds directed toward the H4 backbone but does not interact with the nearby chlorophenyl group of JQ1. Competitive experiments involving JQ1 and tetra-acetylated H4 were performed with this mixed profile group. They showed that JQ1 was a better inhibitor of the interaction of BRD4 with the H4 peptide when Leu-94 and Asp-145 were mutated to alanine, whereas it showed similar inhibition for I146A or a slightly decreased one for P82A in comparison to the wild-type protein.

The third group we could define includes Asp-144 and Tyr-139. For both these mutated residues, the JQ1 and histone binding affinities were only slightly altered in comparison to the wild-type protein, indicating a marginal role in binding of either partner. Interestingly, the BD1 residue Asp-144 is replaced by histidine in BD2, which can affect both the overall structure and the local charge and possibly account for the different function of these bromodomains.

Overall our results provide a deeper understanding of the role of individual amino acids in histone and inhibitor recognition. They identify the key amino acids that inhibitors like JQ1 need to address for efficient inhibition of BRD4 and also reveal that residues with a mixed profile between loss of binding to histone and to JQ1 exist. The data also show that different kinds of interactions take place, suggesting that resistance mutations compatible with BRD4 function are possible. Future analysis of clinical samples will show whether such mutations occur in patients treated with BET inhibitors.

## Footnotes

3 The abbreviations used are:

BET: bromodomain and extra-terminal domain
BRD4: bromodomain protein 4
H3: histone 3
H4: histone 4
NMC: NUT (nuclear protein in testis) midline carcinoma
SPR: surface plasmon resonance
TSA: thermal shift assay
FP: fluorescence polarization
TR-FRET: time-resolved fluorescence resonance energy transfer
FRAP: fluorescence recovery after photobleaching
BG: background
TAMRA: tetramethylrhodamine
RU: response units.

## References

[1] Berger S. L. (2007) The complex language of chromatin regulation during transcription. Nature 447, 407–412 17522673 10.1038/nature05915

[2] Bannister A. J. Kouzarides T. (2011) Regulation of chromatin by histone modifications. Cell Res. 21, 381–395 21321607 10.1038/cr.2011.22 PMC3193420

[3] Zeng L. Zhou M. M. (2002) Bromodomain. An acetyl-lysine binding domain. FEBS Lett. 513, 124–128 11911891 10.1016/s0014-5793(01)03309-9

[4] Filippakopoulos P. Picaud S. Mangos M. Keates T. Lambert J. P. Barsyte-Lovejoy D. Felletar I. Volkmer R. Müller S. Pawson T. Gingras A. C. Arrowsmith C. H. Knapp S. (2012) Histone recognition and large-scale structural analysis of the human bromodomain family. Cell 149, 214–231 22464331 10.1016/j.cell.2012.02.013 PMC3326523

[5] Florence B. Faller D. V. (2001) You bet-cha. A novel family of transcriptional regulators. Front Biosci. 6, D1008–D1018 11487468 10.2741/florence

[6] Vidler L. R. Brown N. Knapp S. Hoelder S. (2012) Druggability analysis and structural classification of bromodomain acetyl-lysine binding sites. J. Med. Chem. 55, 7346–7359 22788793 10.1021/jm300346w PMC3441041

[7] Wang R. Li Q. Helfer C. M. Jiao J. You J. (2012) The bromodomain protein Brd4 associated with acetylated chromatin is important for maintenance of higher-order chromatin structure. J. Biol. Chem. 287, 10738–10752 22334664 10.1074/jbc.M111.323493 PMC3322821

[8] Jang M. K. Mochizuki K. Zhou M. Jeong H. S. Brady J. N. Ozato K. (2005) The bromodomain protein Brd4 is a positive regulatory component of P-TEFb and stimulates RNA polymerase II-dependent transcription. Mol. Cell 19, 523–534 16109376 10.1016/j.molcel.2005.06.027

[9] Schröder S. Cho S. Zeng L. Zhang Q. Kaehlcke K. Mak L. Lau J. Bisgrove D. Schnölzer M. Verdin E. Zhou M. M. Ott M. (2012) Two-pronged binding with bromodomain-containing protein 4 liberates positive transcription elongation factor b from inactive ribonucleoprotein complexes. J. Biol. Chem. 287, 1090–1099 22084242 10.1074/jbc.M111.282855 PMC3256921

[10] Yang Z. He N. Zhou Q. (2008) Brd4 recruits P-TEFb to chromosomes at late mitosis to promote G_1_ gene expression and cell cycle progression. Mol. Cell. Biol. 28, 967–976 18039861 10.1128/MCB.01020-07 PMC2223388

[11] Chiang C. M. (2009) Brd4 engagement from chromatin targeting to transcriptional regulation. Selective contact with acetylated histone H3 and H4. F1000 Biol. Rep. 1, 98 20495683 10.3410/B1-98 PMC2873783

[12] Zhang W. Prakash C. Sum C. Gong Y. Li Y. Kwok J. J. Thiessen N. Pettersson S. Jones S. J. Knapp S. Yang H. Chin K. C. (2012) Bromodomain-containing protein 4 (BRD4) regulates RNA polymerase II serine 2 phosphorylation in human CD4+ T cells. J. Biol. Chem. 287, 43137–43155 23086925 10.1074/jbc.M112.413047 PMC3522308

[13] Zhang G. Liu R. Zhong Y. Plotnikov A. N. Zhang W. Zeng L. Rusinova E. Gerona-Nevarro G. Moshkina N. Joshua J. Chuang P. Y. Ohlmeyer M. He J. C. Zhou M. M. (2012) Down-regulation of NF-κB transcriptional activity in HIV-associated kidney disease by BRD4 inhibition. J. Biol. Chem. 287, 28840–28851 22645123 10.1074/jbc.M112.359505 PMC3436579

[14] Zou Z. Huang B. Wu X. Zhang H. Qi J. Bradner J. Nair S. Chen L. F. (2013) Brd4 maintains constitutively active NF-κB in cancer cells by binding to acetylated RelA. Oncogene 10.1038/onc.2013.179 PMC3913736 23686307

[15] Rahman S. Sowa M. E. Ottinger M. Smith J. A. Shi Y. Harper J. W. Howley P. M. (2011) The Brd4 extraterminal domain confers transcription activation independent of pTEFb by recruiting multiple proteins, including NSD3. Mol. Cell. Biol. 31, 2641–2652 21555454 10.1128/MCB.01341-10 PMC3133372

[16] Oliver S. S. Denu J. M. (2011) Disrupting the reader of histone language. Angew Chem. Int. Ed. Engl. 50, 5801–5803 21618372 10.1002/anie.201101414 PMC3327163

[17] Filippakopoulos P. Qi J. Picaud S. Shen Y. Smith W. B. Fedorov O. Morse E. M. Keates T. Hickman T. T. Felletar I. Philpott M. Munro S. McKeown M. R. Wang Y. Christie A. L. West N. Cameron M. J. Schwartz B. Heightman T. D. La Thangue N. French C. A. Wiest O. Kung A. L. Knapp S. Bradner J. E. (2010) Selective inhibition of BET bromodomains. Nature 468, 1067–1073 20871596 10.1038/nature09504 PMC3010259

[18] Nicodeme E. Jeffrey K. L. Schaefer U. Beinke S. Dewell S. Chung C. W. Chandwani R. Marazzi I. Wilson P. Coste H. White J. Kirilovsky J. Rice C. M. Lora J. M. Prinjha R. K. Lee K. Tarakhovsky A. (2010) Suppression of inflammation by a synthetic histone mimic. Nature 468, 1119–1123 21068722 10.1038/nature09589 PMC5415086

[19] Dawson M. A. Prinjha R. K. Dittmann A. Giotopoulos G. Bantscheff M. Chan W. I. Robson S. C. Chung C. W. Hopf C. Savitski M. M. Huthmacher C. Gudgin E. Lugo D. Beinke S. Chapman T. D. Roberts E. J. Soden P. E. Auger K. R. Mirguet O. Doehner K. Delwel R. Burnett A. K. Jeffrey P. Drewes G. Lee K. Huntly B. J. Kouzarides T. (2011) Inhibition of BET recruitment to chromatin as an effective treatment for MLL-fusion leukaemia. Nature 478, 529–533 21964340 10.1038/nature10509 PMC3679520

[20] Delmore J. E. Issa G. C. Lemieux M. E. Rahl P. B. Shi J. Jacobs H. M. Kastritis E. Gilpatrick T. Paranal R. M. Qi J. Chesi M. Schinzel A. C. McKeown M. R. Heffernan T. P. Vakoc C. R. Bergsagel P. L. Ghobrial I. M. Richardson P. G. Young R. A. Hahn W. C. Anderson K. C. Kung A. L. Bradner J. E. Mitsiades C. S. (2011) BET bromodomain inhibition as a therapeutic strategy to target c-Myc. Cell 146, 904–917 21889194 10.1016/j.cell.2011.08.017 PMC3187920

[21] Mertz J. A. Conery A. R. Bryant B. M. Sandy P. Balasubramanian S. Mele D. A. Bergeron L. Sims R. J. 3rd. (2011) Targeting MYC dependence in cancer by inhibiting BET bromodomains. Proc. Natl. Acad. Sci. U.S.A. 108, 16669–16674 21949397 10.1073/pnas.1108190108 PMC3189078

[22] Lovén J. Hoke H. A. Lin C. Y. Lau A. Orlando D. A. Vakoc C. R. Bradner J. E. Lee T. I. Young R. A. (2013) Selective inhibition of tumor oncogenes by disruption of super-enhancers. Cell 153, 320–334 23582323 10.1016/j.cell.2013.03.036 PMC3760967

[23] Wu S. Y. Lee A. Y. Lai H. T. Zhang H. Chiang C. M. (2013) Phospho switch triggers Brd4 chromatin binding and activator recruitment for gene-specific targeting. Mol. Cell 49, 843–857 23317504 10.1016/j.molcel.2012.12.006 PMC3595396

[24] French C. A. Miyoshi I. Aster J. C. Kubonishi I. Kroll T. G. Dal Cin P. Vargas S. O. Perez-Atayde A. R. Fletcher J. A. (2001) BRD4 bromodomain gene rearrangement in aggressive carcinoma with translocation t(15;19). Am. J. Pathol. 159, 1987–1992 11733348 10.1016/S0002-9440(10)63049-0 PMC1850578

[25] French C. A. Miyoshi I. Kubonishi I. Grier H. E. Perez-Atayde A. R. Fletcher J. A. (2003) BRD4-NUT fusion oncogene. A novel mechanism in aggressive carcinoma. Cancer Res. 63, 304–307 12543779

[26] French C. A. (2013) The importance of diagnosing NUT midline carcinoma. Head Neck Pathol. 7, 11–16 23463074 10.1007/s12105-013-0428-1 PMC3597165

[27] Herrmann H. Blatt K. Shi J. Gleixner K. V. Cerny-Reiterer S. Müllauer L. Vakoc C. R. Sperr W. R. Horny H. P. Bradner J. E. Zuber J. Valent P. (2012) Small-molecule inhibition of BRD4 as a new potent approach to eliminate leukemic stem and progenitor cells in acute myeloid leukemia AML. Oncotarget. 3, 1588–1599 23249862 10.18632/oncotarget.733 PMC3681497

[28] Da Costa D. Agathanggelou A. Perry T. Weston V. Petermann E. Zlatanou A. Oldreive C. Wei W. Stewart G. Longman J. Smith E. Kearns P. Knapp S. Stankovic T. (2013) BET inhibition as a single or combined therapeutic approach in primary paediatric B-precursor acute lymphoblastic leukaemia. Blood Cancer J. 3, e126 23872705 10.1038/bcj.2013.24 PMC3730202

[29] Zuber J. Shi J. Wang E. Rappaport A. R. Herrmann H. Sison E. A. Magoon D. Qi J. Blatt K. Wunderlich M. Taylor M. J. Johns C. Chicas A. Mulloy J. C. Kogan S. C. Brown P. Valent P. Bradner J. E. Lowe S. W. Vakoc C. R. (2011) RNAi screen identifies Brd4 as a therapeutic target in acute myeloid leukaemia. Nature 478, 524–528 21814200 10.1038/nature10334 PMC3328300

[30] Puissant A. Frumm S. M. Alexe G. Bassil C. F. Qi J. Chanthery Y. H. Nekritz E. A. Zeid R. Gustafson W. C. Greninger P. Garnett M. J. McDermott U. Benes C. H. Kung A. L. Weiss W. A. Bradner J. E. Stegmaier K. (2013) Targeting MYCN in neuroblastoma by BET bromodomain inhibition. Cancer Discov. 3, 308–323 23430699 10.1158/2159-8290.CD-12-0418 PMC3672953

[31] Wyce A. Ganji G. Smitheman K. N. Chung C. W. Korenchuk S. Bai Y. Barbash O. Le B. Craggs P. D. McCabe M. T. Kennedy-Wilson K. M. Sanchez L. V. Gosmini R. L. Parr N. McHugh C. F. Dhanak D. Prinjha R. K. Auger K. R. Tummino P. J. (2013) BET inhibition silences expression of MYCN and BCL2 and induces cytotoxicity in neuroblastoma tumor models. PLoS ONE 8, e72967 24009722 10.1371/journal.pone.0072967 PMC3751846

[32] Lockwood W. W. Zejnullahu K. Bradner J. E. Varmus H. (2012) Sensitivity of human lung adenocarcinoma cell lines to targeted inhibition of BET epigenetic signaling proteins. Proc. Natl. Acad. Sci. U.S.A. 109, 19408–19413 23129625 10.1073/pnas.1216363109 PMC3511085

[33] Shimamura T. Chen Z. Soucheray M. Carretero J. Kikuchi E. Tchaicha J. H. Gao Y. Cheng K. A. Cohoon T. J. Qi J. Akbay E. Kimmelman A. C. Kung A. L. Bradner J. E. Wong K. K. (2013) Efficacy of BET bromodomain inhibition in Kras-mutant non-small cell lung cancer. Clin. Cancer Res. 19, 6183–6192 24045185 10.1158/1078-0432.CCR-12-3904 PMC3838895

[34] Segura M. F. Fontanals-Cirera B. Gaziel-Sovran A. Guijarro M. V. Hanniford D. Zhang G. González-Gomez P. Morante M. Jubierre L. Zhang W. Darvishian F. Ohlmeyer M. Osman I. Zhou M. M. Hernando E. (2013) BRD4 sustains proliferation and represents a new target for epigenetic therapy in melanoma. Cancer Res. 73, 6264–6276 23950209 10.1158/0008-5472.CAN-13-0122-T PMC4254777

[35] Weidner-Glunde M. Ottinger M. Schulz T. F. (2010) WHAT do viruses BET on? Front. Biosci. 15, 537–549 10.2741/3632 20036832

[36] Dhalluin C. Carlson J. E. Zeng L. He C. Aggarwal A. K. Zhou M. M. (1999) Structure and ligand of a histone acetyltransferase bromodomain. Nature 399, 491–496 10365964 10.1038/20974

[37] Owen D. J. Ornaghi P. Yang J. C. Lowe N. Evans P. R. Ballario P. Neuhaus D. Filetici P. Travers A. A. (2000) The structural basis for the recognition of acetylated histone H4 by the bromodomain of histone acetyltransferase gcn5p. EMBO J. 19, 6141–6149 11080160 10.1093/emboj/19.22.6141 PMC305837

[38] Umehara T. Nakamura Y. Jang M. K. Nakano K. Tanaka A. Ozato K. Padmanabhan B. Yokoyama S. (2010) Structural basis for acetylated histone H4 recognition by the human BRD2 bromodomain. J. Biol. Chem. 285, 7610–7618 20048151 10.1074/jbc.M109.062422 PMC2844208

[39] Vollmuth F. Blankenfeldt W. Geyer M. (2009) Structures of the dual bromodomains of the P-TEFb-activating protein Brd4 at atomic resolution. J. Biol. Chem. 284, 36547–36556 19828451 10.1074/jbc.M109.033712 PMC2794770

[40] Dey A. Chitsaz F. Abbasi A. Misteli T. Ozato K. (2003) The double bromodomain protein Brd4 binds to acetylated chromatin during interphase and mitosis. Proc. Natl. Acad. Sci. U.S.A. 100, 8758–8763 12840145 10.1073/pnas.1433065100 PMC166386

[41] Ito T. Umehara T. Sasaki K. Nakamura Y. Nishino N. Terada T. Shirouzu M. Padmanabhan B. Yokoyama S. Ito A. Yoshida M. (2011) Real-time imaging of histone H4K12-specific acetylation determines the modes of action of histone deacetylase and bromodomain inhibitors. Chem. Biol. 18, 495–507 21513886 10.1016/j.chembiol.2011.02.009

[42] Kanno T. Kanno Y. Siegel R. M. Jang M. K. Lenardo M. J. Ozato K. (2004) Selective recognition of acetylated histones by bromodomain proteins visualized in living cells. Mol. Cell 13, 33–43 14731392 10.1016/s1097-2765(03)00482-9

[43] Morinière J. Rousseaux S. Steuerwald U. Soler-López M. Curtet S. Vitte A. L. Govin J. Gaucher J. Sadoul K. Hart D. J. Krijgsveld J. Khochbin S. Müller C. W. Petosa C. (2009) Cooperative binding of two acetylation marks on a histone tail by a single bromodomain. Nature 461, 664–668 19794495 10.1038/nature08397

[44] Pivot-Pajot C. Caron C. Govin J. Vion A. Rousseaux S. Khochbin S. (2003) Acetylation-dependent chromatin reorganization by BRDT, a testis-specific bromodomain-containing protein. Mol. Cell. Biol. 23, 5354–5365 12861021 10.1128/MCB.23.15.5354-5365.2003 PMC165724

[45] Gamsjaeger R. Webb S. R. Lamonica J. M. Billin A. Blobel G. A. Mackay J. P. (2011) Structural basis and specificity of acetylated transcription factor GATA1 recognition by BET family bromodomain protein Brd3. Mol. Cell. Biol. 31, 2632–2640 21555453 10.1128/MCB.05413-11 PMC3133386

[46] Sierra J. R. Cepero V. Giordano S. (2010) Molecular mechanisms of acquired resistance to tyrosine kinase targeted therapy. Mol. Cancer 9, 75 20385023 10.1186/1476-4598-9-75 PMC2864216

[47] Zhang J. Yang P. L. Gray N. S. (2009) Targeting cancer with small molecule kinase inhibitors. Nat. Rev. Cancer 9, 28–39 19104514 10.1038/nrc2559 PMC12406740

[48] Yun C. H. Mengwasser K. E. Toms A. V. Woo M. S. Greulich H. Wong K. K. Meyerson M. Eck M. J. (2008) The T790M mutation in EGFR kinase causes drug resistance by increasing the affinity for ATP. Proc. Natl. Acad. Sci. U.S.A. 105, 2070–2075 18227510 10.1073/pnas.0709662105 PMC2538882

[49] Durocher Y. Perret S. Kamen A. (2002) High-level and high-throughput recombinant protein production by transient transfection of suspension-growing human 293-EBNA1 cells. Nucleic Acids Res. 30, E9 11788735 10.1093/nar/30.2.e9 PMC99848

[50] Barik S. (1993) Site-directed mutagenesis by double polymerase chain reaction. Megaprimer method. Methods Mol. Biol. 15, 277–286 21400286 10.1385/0-89603-244-2:277

[51] Brandts J. F. Lin L. N. (1990) Study of strong to ultratight protein interactions using differential scanning calorimetry. Biochemistry 29, 6927–6940 2204424 10.1021/bi00481a024

[52] Myers J. K. Pace C. N. Scholtz J. M. (1995) Denaturant m values and heat capacity changes. Relation to changes in accessible surface areas of protein unfolding. Protein Sci. 4, 2138–2148 8535251 10.1002/pro.5560041020 PMC2142997

[53] Layton C. J. Hellinga H. W. (2010) Thermodynamic analysis of ligand-induced changes in protein thermal unfolding applied to high-throughput determination of ligand affinities with extrinsic fluorescent dyes. Biochemistry 49, 10831–10841 21050007 10.1021/bi101414z

[54] Cheng Y. Prusoff W. H. (1973) Relationship between the inhibition constant (*K*_1_) and the concentration of inhibitor which causes 50 per cent inhibition (I_50_) of an enzymatic reaction. Biochem. Pharmacol. 22, 3099–3108 4202581 10.1016/0006-2952(73)90196-2

[55] Picaud S. Da Costa D. Thanasopoulou A. Filippakopoulos P. Fish P. V. Philpott M. Fedorov O. Brennan P. Bunnage M. E. Owen D. R. Bradner J. E. Taniere P. O'Sullivan B. Müller S. Schwaller J. Stankovic T. Knapp S. (2013) PFI-1. A highly selective protein interaction inhibitor targeting BET bromodomains. Cancer Res. 73, 3336–3346 23576556 10.1158/0008-5472.CAN-12-3292 PMC3673830

[56] Phair R. D. Gorski S. A. Misteli T. (2004) Measurement of dynamic protein binding to chromatin *in vivo*, using photobleaching microscopy. Methods Enzymol. 375, 393–414 14870680 10.1016/s0076-6879(03)75025-3

[57] Huang B. Yang X. D. Zhou M. M. Ozato K. Chen L. F. (2009) Brd4 coactivates transcriptional activation of NF-κB via specific binding to acetylated RelA. Mol. Cell. Biol. 29, 1375–1387 19103749 10.1128/MCB.01365-08 PMC2643823

[58] Chung C. W. Witherington J. (2011) Progress in the discovery of small-molecule inhibitors of bromodomain-histone interactions. J. Biomol. Screen 16, 1170–1185 21956175 10.1177/1087057111421372

[59] Matulis D. Kranz J. K. Salemme F. R. Todd M. J. (2005) Thermodynamic stability of carbonic anhydrase. Measurements of binding affinity and stoichiometry using ThermoFluor. Biochemistry 44, 5258–5266 15794662 10.1021/bi048135v

[60] Martinez Molina D. Jafari R. Ignatushchenko M. Seki T. Larsson E. A. Dan C. Sreekumar L. Cao Y. Nordlund P. (2013) Monitoring drug target engagement in cells and tissues using the cellular thermal shift assay. Science 341, 84–87 23828940 10.1126/science.1233606

[61] Plass C. Pfister S. M. Lindroth A. M. Bogatyrova O. Claus R. Lichter P. (2013) Mutations in regulators of the epigenome and their connections to global chromatin patterns in cancer. Nat. Rev. Genet. 14, 765–780 24105274 10.1038/nrg3554

[62] Bjerke L. Mackay A. Nandhabalan M. Burford A. Jury A. Popov S. Bax D. A. Carvalho D. Taylor K. R. Vinci M. Bajrami I. McGonnell I. M. Lord C. J. Reis R. M. Hargrave D. Ashworth A. Workman P. Jones C. (2013) Histone H3.3 mutations drive pediatric glioblastoma through up-regulation of MYCN. Cancer Discov. 3, 512–519 10.1158/2159-8290.CD-12-0426 PMC3763966 23539269

[63] Hewings D. S. Rooney T. P. Jennings L. E. Hay D. A. Schofield C. J. Brennan P. E. Knapp S. Conway S. J. (2012) Progress in the development and application of small molecule inhibitors of bromodomain-acetyl-lysine interactions. J. Med. Chem. 55, 9393–9413 22924434 10.1021/jm300915b

[64] Muller S. Filippakopoulos P. Knapp S. (2011) Bromodomains as therapeutic targets. Expert Rev. Mol. Med. 13, e29 21933453 10.1017/S1462399411001992 PMC3177561

[65] Steiner S. Magno A. Huang D. Caflisch A. (2013) Does bromodomain flexibility influence histone recognition? FEBS Lett. 587, 2158–2163 23711371 10.1016/j.febslet.2013.05.032

[66] Sun H. Liu J. Zhang J. Shen W. Huang H. Xu C. Dai H. Wu J. Shi Y. (2007) Solution structure of BRD7 bromodomain and its interaction with acetylated peptides from histone H3 and H4. Biochem. Biophys. Res. Commun. 358, 435–441 17498659 10.1016/j.bbrc.2007.04.139

[67] Prinjha R. K. Witherington J. Lee K. (2012) Place your BETs. The therapeutic potential of bromodomains. Trends Pharmacol. Sci. 33, 146–153 22277300 10.1016/j.tips.2011.12.002

